# Cow’s milk compared to oat drink and its implications for lipid profile– a pilot randomized controlled trial

**DOI:** 10.1186/s12937-026-01314-w

**Published:** 2026-03-18

**Authors:** Hanne Rosendahl-Riise, Eline Olsen Gravrok, Sarah Louise Rung, Jutta Dierkes, Inger Aakre

**Affiliations:** 1 Centre for Nutrition, Department of Clinical Medicine, University of Bergen, Jonas Lies vei 91, Bergen, N-5020 Norway; 2 Department of Clinical Medicine, University of Bergen, Bergen, Norway; 3 Institute of Marine Research, Bergen, Norway

**Keywords:** Oat drink, Cow’s milk, LDL-cholesterol, Pilot randomized controlled trial

## Abstract

A recent dietary trend involves replacing cow’s milk with plant-based drink alternatives. This shift is motivated by the perception that plant-based options are healthier and more environmentally sustainable. One of the most popular options is oat drink; however, the health effects of consuming oat drink as a replacement for cow’ s milk are not well studied. The EFSA health claims state that beta-glucans in oat drink have beneficial effects on cholesterol; however, consumption of oat drink at doses aligned with food-based guidelines for milk and dairy products has not been examined in real-world scenarios.

Thus, the primary goal of this pilot randomized controlled trial was to assess the effectiveness of consuming 600 mL of oat drink daily versus 600 mL of cow’s milk daily over four weeks on blood lipids (total cholesterol, LDL-cholesterol, HDL-cholesterol, and triglycerides) in healthy young women in a real-world setting. A four-week, open-label, parallel-arm randomized controlled trial was conducted on women aged 18–40 years who habitually drink cow’s milk. Participants were randomized 1:1 to consume either 600 mL/day of oat drink or 600 mL/day of semi-skimmed cow’s milk. Two baseline visits were conducted before the intervention, during which informed consent, anthropometric measurements, 24-hour recalls, and blood samples were collected. During the intervention, participants recorded their milk intake in compliance diaries. Two final visits were held at the end of the study, where dietary recalls, anthropometry, and blood samples were repeated.

Of the 33 women enrolled, 32 completed the trial. Baseline characteristics were similar across groups, including age (median 23 years), BMI (median 22. 9 kg/m ²), and dietary intake. Over the four weeks, the oat drink group experienced a significant reduction in mean total cholesterol (-0.3 mmol/L (95% CI 0.6 to 0.0; p = 0.026) and LDL-cholesterol (–0.4 mmol/L (95% CI − 0.6 to − 0. 2; p = 0.002), compared to the cow’ s milk control group. No differences were observed between the groups in HDL- cholesterol, or triglycerides.

In conclusion, these results indicate that replacing cow’s milk with oat drink may lead to modest reductions in total and LDL‑cholesterol over four weeks in healthy young women under real-world conditions. The compliance and feasibility of this effectiveness study were good, with high compliance rates. ClinicalTrials.gov NCT06764173.

**Supplementary Information:**

The online version contains supplementary material available at 10.1186/s12937-026-01314-w.

## Introduction

In recent years, dietary patterns have increasingly shifted towards plant-based alternatives, driven by growing consumer interest in sustainability, ethical considerations, and perceived health benefits [1]. An example of this trend is the rising consumption of plant-based beverages, which has coincided with a decline in traditional cow’s milk sales [2]. Among these alternatives, oat drinks have emerged as one of the fastest-growing options [1]. Compared to dairy and almond drinks, production of oat-based beverages requires substantially less water and generates lower greenhouse gas emissions [3]. While the environmental advantages of plant-based drinks are well-documented, their nutritional implications remain less clear, with limited research addressing potential health effects [4].

Cow’s milk has historically held a central role in Nordic diets and continues to be a culturally significant and nutritionally important component [5]. Cow’s milk is nutrient-dense and, in Norway, national dietary guidelines recommend three daily servings of dairy products including low-fat milk to support adequate calcium and iodine intake [6]. In contrast, oat drinks are typically composed of oats and water, with some formulations including added vegetable oils and micronutrient fortifiers [5]. Their nutritional composition varies considerably depending on processing and fortification practices. Compared to cow’s milk, oat drinks generally contain less protein (and low amounts of one or more essential amino acids) and fewer micronutrients (unless fortified) but offer polyunsaturated fatty acids (from added rapeseed oil) and dietary fibre, primarily in the form of water-soluble, mixed-linkage β-glucan [7, 8].

Current dietary guidelines, including those outlined in the Nordic Nutrition Recommendations (NNR2023), advocate for reduced intake of saturated fat. In Nordic and Baltic diets, milk and dairy products contribute approximately half of total saturated fat intake [9]. Saturated fat is a well-established determinant of plasma total and LDL-cholesterol concentrations and is causally linked to atherosclerotic cardiovascular disease [10, 11]. Conversely, β-glucan from oats has demonstrated lipid-lowering properties and is recognised by the European Food Safety Authority (EFSA) for its role in reducing LDL-cholesterol [12]. Its mechanism involves increased viscosity in the gastrointestinal tract, which impairs cholesterol absorption and enhances excretion. A daily intake of at least 3 g of β-glucan is required to elicit cholesterol-lowering effects in normo- and hypercholesterolemic individuals, typically observable within a few weeks. It should be noted that the cholesterol‑lowering effect of β‑glucan depends not only on a daily intake of 3 g, but also on its molecular weight and resulting viscosity [13].

Demonstrating the effects of replacing cow’s milk with oat drink under tightly controlled dietary conditions—such as full regulation of the background diet—do not reflect the complexity of real-world dietary behaviour. Assessing how oat drink performs when integrated into individuals’ habitual eating patterns is therefore essential for informing evidence-based public health recommendations. To our knowledge, no study has yet evaluated the real-world effectiveness of incorporating oat drink into the everyday diet without controlling for other dietary components. Thus, the primary goal of this pilot randomized controlled trial was to assess the effects of consuming 600 mL of oat drink daily versus 600 mL of cow’s milk daily over four weeks on blood lipids (total cholesterol, LDL-cholesterol, HDL-cholesterol, and triglycerides) in healthy young women in a real-world setting. In addition, the feasibility of drinking the required amount of milk was investigated, as was the iodine nutrition of the participants.

## Methods

### Study design

This study was designed as an effectiveness trial with and open-label randomized, controlled trial (RCT) with a parallel arm structure. The two arms consisted of an intervention arm (oat drink) and a control arm (cow´s milk). Both arms were instructed to drink a minimum of 600 mL of their assigned milk daily. The amount was chosen based on the recommended amount of cow's milk or diary in the Norwegian food-based dietary guidelines [14].The intervention period was four weeks, with four study visits in total: two at baseline and two at the end study. All study visits were held at the Research Unit for Health Services (RUHS) in Bergen, Norway.

### Participants

Participants in this trial were young, healthy women residing in Bergen, Norway, at the time of intervention. Inclusion criteria were women aged 18–40 years who were in good general health. The participants were required to be habitual cow’s milk drinkers. Young women were selected as the target group because they represent the demographic group in Norway most likely to shift from cow’s milk to plant‑based beverages, a transition that may have important nutritional consequences, including reduced iodine intake. Inclusion and exclusion criteria are outlined in Table 1.

**Table 1:** Inclusion and exclusion criteria for the pilot randomized clinical trial

Inclusion criteria	Exclusion criteria
Women aged 18–40 years	Pregnant or lactating
Habitual cow’s milk drinkers	Planning to conceive
Good general health	Known thyroid disease
Signed informed consent	Medication affecting lipid metabolism

### Enrolment

Convenience sampling was used to recruit participants to the study. Recruitment took place predominantly among students in Bergen between August and October 2024. Participants were invited to the study through posters on the University of Bergen campus and through social media platforms. Those willing to participate contacted the study personnel through a digital form. Eligible participants were invited to an initial study visit, where they were provided with detailed information about the study. Informed consent was collected from all participants. 

### Randomization

Participants were randomized into either the intervention (oat milk) or control arm (cow's milk) using block-randomization with a block size of four to ensure balanced group sizes. Randomization was performed in Microsoft Excel using the random number function (RAND). All participant ID-numbers were listed in ascending order and divided into blocks of four. Each block was then assigned a random number between one and six, corresponding to one of six possible sequences of two intervention (A) and two control (B) assignments. This was done to ensure an equal allocation ratio of 1:1. Finally, the assigned sequence was matched with the ID-numbers within each block, deciding each participant’s group allocation.

### Allocation concealment and blinding

The randomization process was done with allocation concealment for the participants, but not the researchers. All participants were assigned to their groups according to the randomized allocation, and no deviations from the randomization sequence occurred. The participants were not informed of their allocation before the second study visit, when starting the intervention, to prevent participants from dropping out due to unwanted group placement. The second visit was within a few days after the initial visit. The trial was open-label as blinding for participants was not feasible, as they would be able to decipher the milk they were drinking by flavour and consistency. Thus, the intervention milk was provided in its original packaging.

### Intervention

The four-week intervention period began after the second study visit. Participants in the intervention arm were instructed to consume 600 mL oat drink daily, while the control group consumed 600 mL cow’s milk, both recording their intake in compliance diaries. Both groups were asked to maintain their regular dietary habits throughout the intervention period. Participants were allowed to consume more oat drink or cow’s milk from their habitual diet if they wished. Additionally, the oat drink arm was not required to abstain from cow's milk and vice versa. The intervention milk could be consumed in any manner the participant preferred, either by drinking or incorporating it into meals, either distributed throughout the day or all-in-one consumption. Supplements containing omega-3s had to be taken at the same dosage and frequency as before the intervention. Participants who did not use such supplements were instructed not to start them during the intervention period. The intervention milk was provided by researchers and collected by participants at the study centre at a weekly interval. Both drink alternatives were regularly bought at the local supermarket and stored cold in refrigerators at the study centre before being handed out to the participants. The provided oat drink and cow milk were “Gryr Naturell Havredrikk” and “Tine lettmelk”. The semi-skimmed cow’s milk was chosen to match the fat content of the oat drink as closely as possible. Their respective nutrient content is outlined in Table 2. ß-glucan content in “Gryr Naturell Havredrikk” was calculated based on the declared content of dietary fiber (0.8 g) and oats (10%/10 g), with an assumption of 4.45 g ß-glucan in 100 g whole oats, as previously found in oats grown in Sweden [15].

**Table 2:** The declared nutrient composition per 100 mL of the iodine-fortified oat drink (intervention) and cow’s milk (control) used in this study

	Oat drink^a^	Cow’s milk^b^
Energy, kcal	48	42
Fat, g	1.5	1.0
Saturated fat, g	0.2	0.6
Protein, g	0.8	3.5
Carbohydrate, g	7.5	4.7
Dietary fibre, g	0.8	-
ß-glucan share of dietary fiber, g^c^	0.56	-
Sugars, g	4.0	4.7
Iodine, µg	16.0	16.9
Calcium, mg	120	119
Vitamin D, µg	1.0	-
Vitamin B12, µg	0.38	0.50
Riboflavin, mg	0.20	0.15
Folic acid, µg	22.0	-
Potassium, mg	-	159
Phosphorus, mg	-	99.7
Biotin, µg	-	5.7

^a^Oat drink provided by GRYR; Gryr Natural Oat Drink

^b^Cow’s milk provided by TINE, 1% semi-skimmed

^c^Calculated ß-glucan share of dietary fiber (per 100 mL) 0.45 g / 0.80 g = 0.56 g

### Data collection

**Fig. 1:**
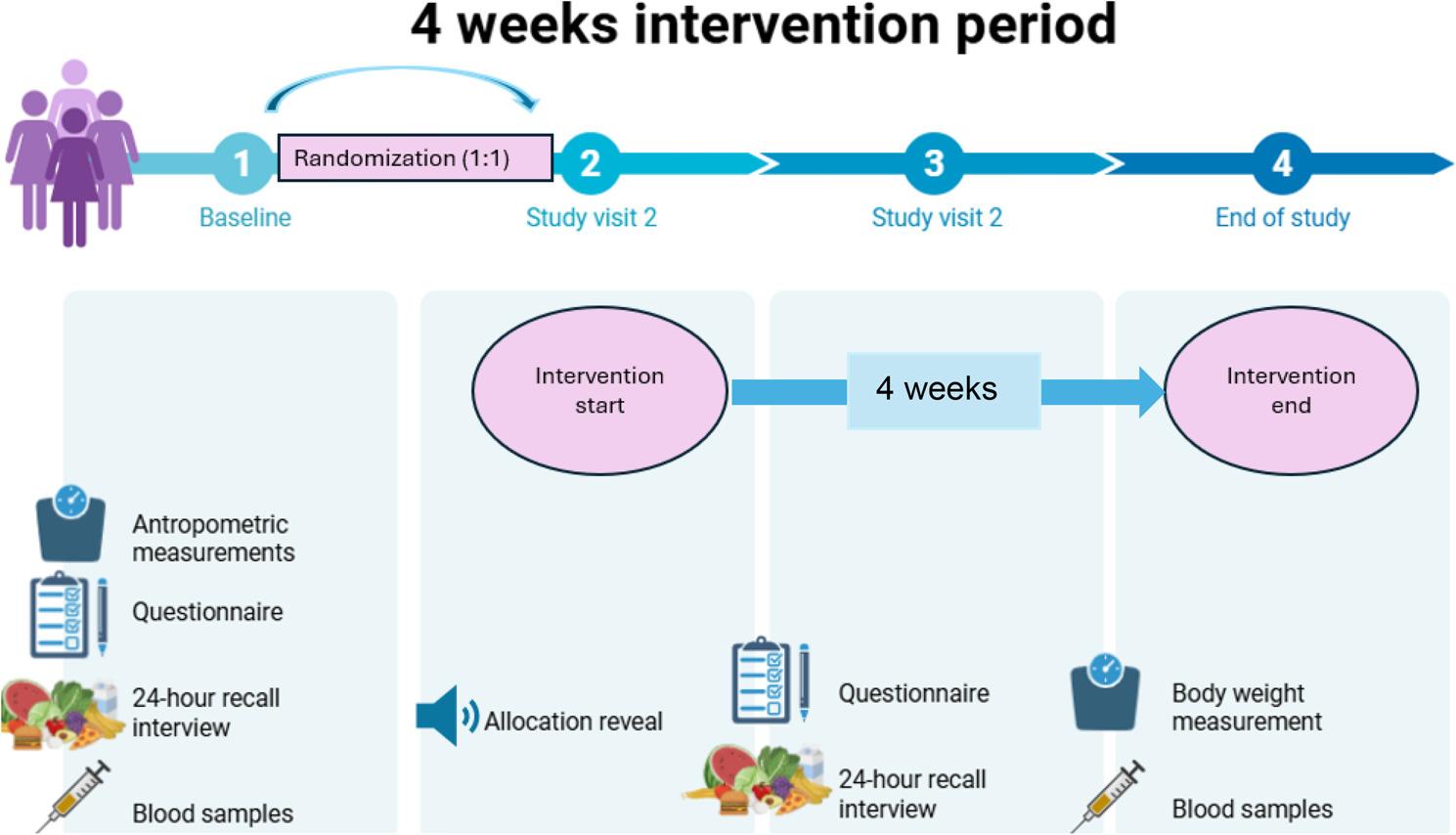
An overview of the timeline and assessment at each timepoint of the 4-week intervention period

An overview of the timeline and procedures at each time point is illustrated in Fig. 1. All data collection was carried out by study personnel at RUHS.

### Clinical assessment and blood samples

#### Blood collection and lipid analysis

Blood samples were collected at the first and fourth study visits from non-fasting participants. Trained personnel at RUHS performed the blood collection using the Vacutainer system. The venous blood was drawn into serum gel-separator tubes to separate serum from plasma and blood cells. The gel-separator tubes were gently inverted a minimum of eight times after sampling before being left for coagulation for a minimum 30 min and a maximum of two hours. The gel tubes were then centrifuged at 2000G for 10 min before one mL of serum was pipetted into test tubes that were stored in a cryobox at -80o Celsius pending analysis. Serum samples were analyzed for total cholesterol (TC), LDL-cholesterol (LDL-C), HDL-cholesterol (HDL-C), and triacylglycerol (TG) through enzymatic colorimetric assays for lipid profile using the COBAS8000 modular analyzer series (Roche Diagnostics). LDL-C was measured directly instead of calculated. Analytical variation for LDL-C was 2.5% (at 3.4 mmol/L), and 3% for TC, HDL-C, and TG. The analysis was performed in the certified laboratory (NSEN-ISO 15189) at Haukeland University Hospital.

#### Laboratory analyses of serum TSH, fT3, fT4, Tg, and TPOAb

The serum blood samples were analysed for TSH, free T3 (fT3), free T4 (fT4), and thyroid peroxidase antibodies (TPOAb) in a certified laboratory (NSEN-ISO 15189) at Haukeland University Hospital, using Electrochemiluminescence Immunoassay (ECLIA). All analyses were performed on the Cobas8000 instrument (the e801 model by Roche). The recorded analytical and intraindividual biological variation rates for TSH, fT3, fT4, Tg, and TPOAb were 5%/19%, 7%/7.9%, 5%/5.7%, 6.5%/13%, 6.4%/8.5%, and 11.3%, respectively.

#### Anthropometric measurements

Anthropometric measurements were performed before and after intervention, following standardized protocols at visit 1 and visit 4. Anthropometric measurements were performed in light clothing without shoes. Bodyweight was measured with a calibrated digital scale (Seca 877, Hamburg, Germany), to the nearest 0.1 kg. Height was measured at study visit 1 using a stadiometer (Seca 217, Hamburg, Germany), rounded to the nearest 0.5 cm, with participants standing upright, feet together, and head in Frankfort horizontal plane. BMI was calculated as weight (kg) divided by height squared (m^2^).

#### Sociodemographic data

Sociodemographic data, including age, education level, and smoking habits, were collected through a standardized interview at the first study visit using a study specific questionnaire. On the third study visit, they were asked to confirm if the reported data was still valid or if any changes had occurred.

#### Dietary assessment

Dietary intake was assessed through two 24-hour recall interviews (24 HR), conducted by master’s students in nutrition with extensive training in conducting 24 HR. The 24 HR was performed in person at baseline (study visit 1) and near endpoint (study visit 3), using the multiple pass method [16] to assess the dietary intake, focusing on dietary sources of macronutrients, fatty acids, iodine, and dietary fibre. The total food and drink intake was entered into “Kostholdsplanleggeren”, a dietary analysis tool developed by the Norwegian Directorate of Health and the Norwegian Food Safety Authority [17]. The tool is based on the Norwegian Food Composition Table [18].

### Assessment of urinary iodine

Twenty‑four–hour urine samples were collected from all 32 participants at baseline and at the end of the study. Participants were instructed to collect all urine over 24 hours, to record the start and end times, and to note any missed voids. Total urine volume was read directly from the graduated collection container. Each 24‑hour sample was homogenised, and aliquots were pipetted into cryovials and stored at − 80 °C until analysis. Two additional aliquots were stored at the local biobank for future research.

Urinary iodine concentrations were analysed at the Institute of Marine Research (IMR) using inductively coupled plasma mass spectrometry (ICP‑MS). Certified reference materials were included in each analytical run to ensure accuracy, and the limit of quantification was 7.8 µg/L. Daily urinary iodine excretion (UIE) was calculated as urinary iodine concentration (µg/L) multiplied by the total 24‑hour urine volume (L). The estimated daily iodine intake was derived by assuming that approximately 90% of dietary iodine is excreted in urine.

### Compliance

Compliance with the intervention was tracked through compliance diaries provided to each participant before starting the intervention. Participants were instructed to log their daily consumption of intervention milk, including volume, date, and time of intake.

### Adverse events

Adverse events (AEs) were monitored throughout the intervention period. Participants were encouraged to contact the research team via email or telephone if they experienced any unexpected symptoms, health concerns, or side effects during or following the intervention and data collection period. In addition, during the weekly milk pick-ups, short debriefing sessions were conducted to inquire about participants’ well-being and to identify any difficulties encountered. In the case of a reported AE, participants were invited for further assessment to determine the severity and potential relatedness to the intervention. AEs were classified by severity as mild, moderate, or severe, and categorized as either related or unrelated to the intervention.

### Sample size

Statistical power was calculated hypothesizing that oat drink would decrease total cholesterol due to the presence of ß-glucan and polyunsaturated fatty acids (PUFAs). The total cholesterol level in the cow’s milk arm was expected to remain unchanged. Total cholesterol was selected as the primary outcome because LDL‑cholesterol constitutes the predominant fraction of total cholesterol, resulting in a strong biological and quantitative correspondence between the two measures. The initial power calculations were not based on accurate data or prior evidence. A post hoc power analysis was subsequently conducted using findings reported by Wolever et al. [19]. The mean difference was set at 0.2 mmol/L, with a standard deviation of 0.3, with an alpha of 0.05, and a power of 80%, the required number per group was 35. This comparison has not previously been tested; therefore, this trial was designed as a pilot to test the feasibility of such a trial. The sample size was limited to 32 participants. The size was considered feasible with general guidance on sample sizes for pilot and feasibility studies [20–25].

### Ethics

This study was approved by the Regional Ethical Committee (REK 712892) and registered in ClinicalTrials.gov (NCT06764173: Study Details | NCT06764173 | The Difference in Health Outcomes After Drinking Cow’s Milk Compared to Oat Milk - a Pilot Randomized Controlled Study | ClinicalTrials.gov, date submitted: 2025-01-02). The study was conducted in accordance with the ethical principles of the Declaration of Helsinki. Participants received both oral and written information regarding the study. Participation was voluntary, and all participants provided signed written consent. Participants could withdraw from the study at any time without providing a reason.

### Statistics

Statistical analyses were performed using SPSS V.30 (SPSS, Inc., IBM Company). Participants who did not complete the intervention and follow-up were excluded from statistical analyses. Missing values were not imputed. All statistical tests were two-tailed, and the statistical significance level was set to *p* < 0.05. To evaluate the effect of the intervention on post‑intervention blood lipid outcomes, we applied analysis of covariance (ANCOVA). For each blood lipid, the post‑intervention value was entered as the dependent variable, the intervention group as the fixed factor, and the baseline value of the blood lipid as a covariate. Age, energy intake at baseline, and change in saturated fat intake were tested as covariates but did not change the estimates significantly. This approach provides adjusted between‑group comparisons and accounts for any baseline imbalance. Estimated marginal means (EMMs) and 95% confidence intervals (CIs) were derived from the model. Assumptions of normality of residuals, homogeneity of variance (Levene’s test), linearity, and homogeneity of regression slopes (group × covariate interaction) were examined and met. However, for the dietary intake data, the data were not normally distributed, therefor the between-group comparisons were performed using Mann–Whitney U tests, with Hodges-Lehmann point estimates and 95% Confidence intervals (CI), while explanatory analysis used within-group comparisons using Wilcoxon signed-rank tests. Categorical baseline variables were compared between groups using Fisher’s Exact Test for tables with expected cell counts ≤ 5, and the Chi-square test when assumptions for expected counts were met.

## Results

### Study population

The participant flow throughout the trial is outlined in Supplementary Fig. 1. Population background characteristics are presented in Table 3. A total of 32 participants, all women, participated in the study. The mean (SD) age was 23 [2] years, and the median (p25-75) BMI at baseline was 22.9 (21.8–24.2) kg/m^2^. None of the participants reported any habits of smoking or sniffing. The baseline characteristics for the intervention and control group were similar between the two groups.

**Table 3:** Baseline characteristics of the study participants within the intervention and control group

	Intervention *n* = 17	Control *n* = 15
Age, years	24.0 (23.0, 24.0)	23.0 (21.5, 25.0)
Weight, kg	65.0 (59.1, 70.2)	67.5 (62.4, 69.1)
BMI, kg/m^2^	23.0 (19.9, 24.3)	22.8 (22.1, 24.2)
Marital status, n %		
Single	13 (76)	12 (80)
Married /cohabiting	4 (24)	3 (20)
Highest educational level, n %		
High school	13 (77)	12 (80)
University	4 (23)	3 (20)
Current nicotine usage, n %	0 (0)	0 (0)
Hormonal contraceptives, n %	7 (41)	7 (47)

Continuous variables are presented as median (p25, p75), categorical variables are presented as n, %

### Compliance

The mean duration of the intervention was 28.4 days for the oat drink group, and 28.6 days for the cow’s milk group. There were no significant differences in daily consumption between the oat drink and cow’s milk groups (*p* = 0.621). Daily intervention drink intake was recorded at 590 mL for all participants and both intervention groups. The range of the daily intake was 540–600 mL for all participants and both groups. The oat milk intake prior to the intervention was overall very low or non-existent across groups. All of the participants consumed cow’s milk before the intervention started. The majority of participants reported that the intake of oat- or cow’s milk during intervention exceeded their habitual intake prior to intervention. With 93.3% reporting increased intake in the control group compared to 58.8% in the intervention group. Additionally, 10 participants (60%) in the intervention group reported that the allocated oat milk replaced some or all of their pre-existing cow’s milk intake.


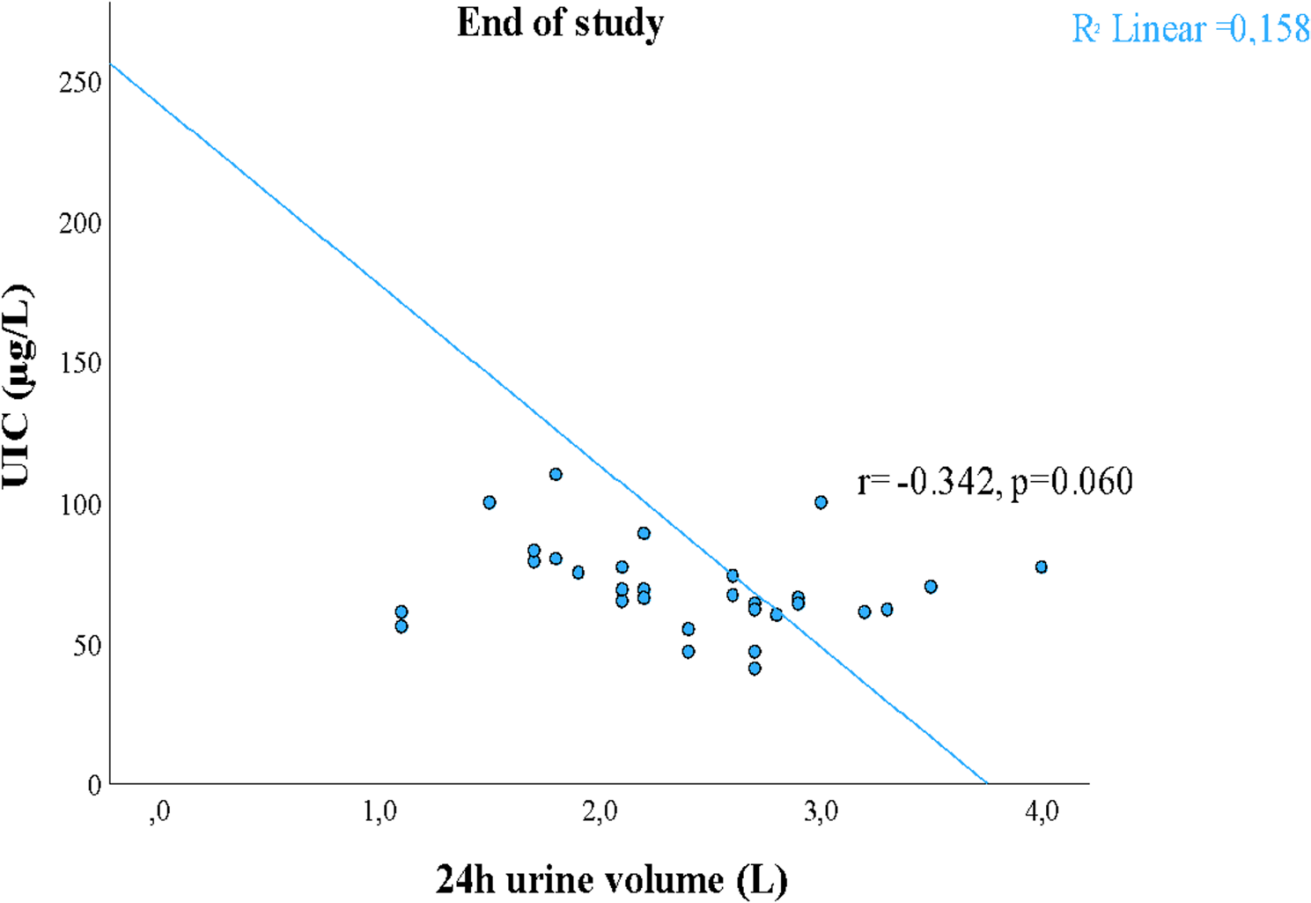


### Lipid concentration

Estimated marginal means of the serum lipid concentrations at the end of the study and the mean group differences between the intervention and control groups are shown in Table 4. The mean difference for TC was − 0.3 mmol/L (95% CI 0.6 to 0.0; *p* = 0.026). The mean difference for LDL-C was − 0.4 mmol/L (95% CI − 0.6 to − 0.1; *p* = 0.002). The mean HDL-C and TG were not different. Adding age, total energy intake at baseline, and change in saturated fat intake as covariates into the model did not change the estimate, with a mean difference for TC of -0.3 mmol/L, 95% CI -0.6 to 0.0, *p*=0.026). 

**Table 4:** Estimated marginal means and mean differences between the intervention group and the control group

Serum lipid mmol/L	EMM (95% CI)^a^	Mean difference (95% CI)^b^	*P* values
Total cholesterol		-0.3 (-0.6—0.04)	0.026
Intervention (*n* = 17)	4.2 (4.0-4.4)		
Control (*n* = 15)	4.5 (4.3–4.7)		
LDL-cholesterol		-0.4 (-0.6—0.1)	0.002
Intervention (*n* = 17)	2.3 (2.2–2.5)		
Control (*n* = 15)	2.7 (2.5–2.8		
HDL-cholesterol		0.04 (-0.1-0.2)	0.538
Intervention (*n* = 17)	1.8 (1.7–1.9)		
Control (*n* = 15)	1.7 (1.6–1.8)		
Triacylglycerol		0.05 (-0.2-0.3)	0.690
Intervention (*n* = 17)	0.9 (0.8–1.1)		
Control (*n* = 15)	0.9 (0.7–1.1)		

*EMM* Estimated marginal means, *95% CI*  95% Confidence interval

^a^blood lipid value at baseline as covariates

^b^mean difference based on the EMM between oat drink and cow’s milk, adjustment for multiple comparisons using Bonferroni

### Estimated dietary intake and body weight

Estimated dietary intake of macronutrients, fatty acids, and dietary fibre from 24 HR at baseline and week 4 is presented in Table 5. At the end of the study, the median carbohydrate intake was lowered by 23 g in the intervention arm versus increased by 38 g in controls. The difference in change between the groups was − 51 g (95% CI − 112 to − 1; *p* = 0.049). Protein intake in the intervention arm changed by + 3 g versus + 20 g in controls. The between-group difference in median change was − 24 g (95% CI − 51 to − 6; *p* = 0.022). Saturated-fat intake changed by − 1 g in the intervention arm versus + 8 g in the control arm. The between-group median difference was − 11 g (95% CI − 26 to − 1; *p* = 0.024). In the intervention arm, the change in dietary cholesterol was + 3 mg/d versus + 177 mg/d in the control arm. The between-group median difference in change was − 203 mg (95% CI − 376 to − 54; *p* = 0.010). No significant between-group differences were observed for change in energy intake, total fat intake, MUFAs, PUFAs, or dietary fibre. The increased median intake of saturated fat could be explained by the increased consumption of cow’s milk, which exceeded their habitual consumption of cow’s milk. However, the increased intake of carbohydrates is more difficult to explain, as both lactose from the milk and the oat milk provided the participants with increased carbohydrates. Some of the participants in the control group also added chocolate powder to the cow’s milk that could also explain some of the increase in carbohydrates in the cow’s milk group.

There were no substantial differences in body weight change between the intervention and controls. Median body weight change was 0.3 kg (p25, p75: 0.1, 0.7) in the intervention arm versus 0.1 kg (0.0, 0.3) in controls, with a BMI of 23.2 and 23.1 kg/m^2^ for the intervention and control group, respectively Between the groups, the median difference was ~ 0 kg (95% CI − 0.6 to 0.4; *p* = 0.823) (data not shown).

**Table 5:** Estimated dietary intake from two 24 h at baseline and 4 weeks, and between-group differences in change

	Baseline	End of study	∆ Baseline-end of study	Between-group ∆^a^	*p*-value
	Median (p25, p75)				
Energy, kcal				-245 (-644, 236)	0.350
Intervention	2460 (1940, 2821)	2660 (2130, 2960)	172 (-425, 574)		
Control	2350 (2000, 2620)	2380 (2170, 3360)	187 (-51.9, 781)		
Carbohydrate, g				-51 (-112, -1)	0.049
Intervention	270 (212, 318)	247 (187, 272)	-23 (-70, 41)		
Control	212 (175, 284)	249 (191, 322)	38 (-18, 72)		
Dietary fibre, g				2 (-8, 11)	0.602
Intervention	27 (22,35)	28 (23, 38)	2 (-7, 14)		
Control	28 (18, 39)	33 (19,39)	1.7 (-7, 5)		
Protein, g				-24 (-51, -6)	0.022
Intervention	94 (75, 111)	95 (74, 118)	3 (-17, 31)		
Control	96 (90, 120)	131 (112, 150)	20 (16, 46)		
Fat, g				-23 (-70, 13)	0.230
Intervention	103 (72, 121)	90 (57, 112)	-12 (-55, 23)		
Control	86 (62, 117)	92 (76, 129)	0 (-16, 44)		
SFA, g				-11 (-26, -1)	0.024
Intervention	36 (24, 47)	27 (16, 42)	-1 (-19,5)		
Control	24 (21, 38)	33 (26, 54)	8 (0, 15)		
MUFAS, g				-4 (-16, 7)	0.576
Intervention	23 (13, 37)	25 (17, 36)	5 (-14, 11)		
Control	24 (21, 35)	28 (26, 39)	4 (-1, 11)		
PUFAS, g				3 (-2, 10)	0.153
Intervention	11 (7, 17)	13 (8, 20)	2.2 (-6, 7)		
Control	14 (8, 16)	13 (9, 16)	1.4 (-9, 4)		
Cholesterol, g				-203 (-376, -54)	0.010
Intervention	166 (88, 443)	169 (110, 370)	3 (-183, 131)		
Control	241 (119, 339)	444 (249, 517)	177 (62, 384)		

Values are median and 25th-75th percentiles (p25, p75)

^a^Between-group Δin intervention and control group, and 95% CI from Hodges–Lehmann estimator (Mann-Whitney U)

*P*-values are two-tailed

### Iodine nutrition

As a safety precaution, the iodine nutrition of the participants was assessed following the intervention. The results can be found in the Supplementary Tables 1 and 2.

### Adverse events

No serious adverse events that required medical attention were reported. One participant in the intervention group experienced a mild adverse event characterized by gastrointestinal symptoms. These included stomach pain and bloating in the epigastric region following oat milk consumption, especially when consumed quickly or in large amounts. The event was classified as mild and related to the intervention. The symptoms resolved on their own, and she did not wish to stop the intervention but opted for two days of non-compliance.

## Discussion

To our knowledge, this pilot randomised controlled effectiveness trial is the first to evaluate the health and nutritional implications of habitual oat drink consumption compared with cow’s milk in a real-life setting, with a particular focus on lipid profiles. After four weeks of intervention, a reduction in TC and LDL-C, d in the oat drink group compared to the cow’s milk group,. No significant differences were found between the groups for HDL-C, TG, or body weight.

### Lipid outcomes and comparison with previous research

To date, no studies have investigated the effects of commercially available oat drinks under real-world consumption conditions. However, soy milk in particular has been more extensively studied than cow’s milk with respect to lipid and cardiometabolic outcomes, and evidence indicates that soy milk not adversely affect established cardiometabolic risk factors and may confer benefits for blood lipids, blood pressure, and inflammatory markers in adults across a range of health statuses [26]. The TC and LDL-C reduction observed in the present trial is broadly consistent with findings from previous studies examining oat-based beverages, which have reported variable effects on total cholesterol, similar reductions in LDL-C, minimal or no changes in HDL-C and TG [19, 27, 28]. However, direct comparisons are limited by methodological differences in the mentioned studies. For example, one study lacks sufficient detail regarding the intervention protocol, making its findings difficult to interpret [27]. Another utilised rice drink as the control rather than cow’s milk, which limits its relevance to typical dietary substitution scenarios [28]. Furthermore, the oat drink used in the third study was specifically formulated to contain high molecular weight β-glucan, differing from the commercially available product used in the present trial. In addition, the participants had borderline high cholesterol at baseline [19]. These distinctions underscore the novelty and practical relevance of the current study, which evaluated a widely available oat drink in a real-life setting.

### Mechanistic considerations and dietary compensation

As this trial was powered to detect between‑group differences in total cholesterol, the observed change in total cholesterol should be interpreted with appropriate emphasis. Although the reduction in LDL‑C paralleled the total cholesterol response, LDL‑C was a secondary outcome, and the study was not powered to detect differences in LDL-C. Thus, while biologically consistent, the LDL‑C findings should be viewed as preliminary. However, as the reduction in total cholesterol was driven primarily by changes in LDL-C, the mechanisms related to LDL metabolism warrant consideration. The LDL-C–lowering effect may be attributable to known mechanisms associated with β-glucan, including increased intestinal viscosity and reduced cholesterol absorption [29]. However, the real-world setting of this trial introduces dietary compensation effects. Participants were instructed to maintain their habitual diet, yet the 24-HR revealed no significant increase in total energy intake, despite the intervention with 600 mL of oat drink or cow’s milk (approx. 250–300 kcal/day). This suggests that participants substituted rather than supplemented their diets. Indeed, 60% of participants in the intervention group reported reducing or eliminating cow’s milk during the trial, indicating that the LDL-C reduction may reflect both the inclusion of oat drink and the exclusion of cow’s milk. Conversely, some control participants reported increased milk consumption, implying that the control group may have undergone a separate dietary shift. These compensatory behaviours complicate interpretation and may attenuate between-group differences.

Despite the oat drink providing 4.8 g of dietary fibre daily, no significant increase in fibre intake was detected, possibly due to the displacement of other fibre-rich foods. Given the high baseline fibre intake, it is plausible that β-glucan replaced other sources of soluble fibre, thereby confounding the attribution of LDL-C reduction solely to the oat drink.

### Satiety, energy intake, and weight stability

β-glucan has been proposed to enhance satiety via the ability to create viscosity, thus delaying gastric emptying, potentially reducing energy intake and lipid levels [29]. However, the satiety effect is dose-dependent [30], and the dose per meal during the trial was likely insuffcient to have an enhanced satiety effect. This is further supported by the fact that energy intake remained similar between the groups, and no differences in body weight were observed. This suggests that satiety effects did not contribute meaningfully to the lipid outcomes. The higher protein content of cow’s milk may have offset any satiety advantage of β-glucan, resulting in similar dietary compensation across groups. This pattern is further reflected in the change in protein intake per kilogram of body weight, which increased from a mean of 1.5 g/kg to 1.9 g/kg in the cow’s‑milk group over the intervention period, while remaining largely unchanged in the oat‑drink group. Weight stability throughout the intervention further supports the conclusion that LDL-C reductions were not driven by changes in adiposity or energy balance, both of which are known to influence lipid metabolism [31].

### Fatty acid substitution and dietary lipid quality

In addition to dietary fiber-related mechanisms, the substitution of cow’s milk with oat drink likely altered the quality of dietary fat intake. Cow’s milk is relatively high in saturated fatty acids (SFA), which are known to elevate LDL-C concentrations [32]. In addition, there was observed an increase in dietary cholesterol intake in the cow’s milk group, likely attributable to the increase cow’s milk intake. However, current dietary guidelines have no longer a specific recommendations for dietary cholesterol, as results on the implications of high dietary cholesterol levels on LDL-C remain uncertain [33]. In contrast, the oat drink used in this trial was formulated with rapeseed oil, rich in monounsaturated (MUFA) and polyunsaturated fatty acids (PUFA), both of which are associated with improved lipid profiles.

The 24-HR indicated an 11 g/day reduction in SFA intake in the intervention group relative to controls, who exhibited an 8 g/day increase. These shifts suggest that the LDL-C reduction may have been driven by both β-glucan and improved fatty acid composition. However, the use of two single 24-HR introduces potential for recall bias and misreporting, e.g., overestimation of protein and energy intake thus may not fully capture habitual intake [34].

### Combined effects and clinical relevance

Taken together, the findings suggest that the observed LDL-C reduction could result from the additive effects of β-glucan and dietary fat substitution. While the magnitude of LDL-C reduction (− 0.40 mmol/L) is modest compared to pharmacological interventions such as statins [35], it may still confer meaningful cardiovascular benefit. A comprehensive meta-analysis [36] has shown that each 1.0 mmol/L reduction in LDL-C is associated with a 20–25% reduction in major vascular events. As a theoretical experiment, ,extrapolating from this, a sustained reduction of 0.3–0.4 mmol/L could hypothetically translate to a 6–10% relative risk reduction over time.

Nevertheless, the findings do not support recommending the replacement of cow’s milk with oat drink for all individuals, particularly in populations at risk of undernutrition due to low protein content.

### Strengths and limitations

This trial has several strengths, including the use of a commercially available products. Adherence and proper implementation are crucial factors in determining the success of an effectiveness trial, and high participant retention and good compliance strengthen our findings [37]. However, limitations must be acknowledged. The researchers who enrolled the participants and performed the statistical analysis had access to the random allocation sequence. We acknowledge that we did not conduct a comprehensive assessment of participants’ habitual diets at baseline, which limits our ability to rule out potential pre‑existing dietary differences that could have interacted with the intervention. Nevertheless, it is important to note that, according to EFSA guidance, background diet is not required to be controlled when evaluating the efficacy of a specific dietary component [38]. Instead, the effect should be demonstrated under the proposed conditions of use within the context of normal dietary patterns. A key limitation is that all lipid measurements were obtained under non‑fasting conditions, and the time since the last meal was not recorded. Given the known postprandial variability—particularly for triglycerides—this may have introduced additional within‑group variation and could partly account for the modest between‑group differences observed. However, within the context of this small pilot RCT, the use of a standardized non‑fasting sampling protocol across all participants reduces the likelihood of systematic bias between groups, even though non‑fasting variability may still have contributed to overall measurement noise [39]. The duration of the trial was not long enough to see any changes in body weight, if there would be on. The β-glucan content was estimated indirectly based on assumed oat composition (4.45 g/100 g from Swedish oats), rather than analytically measured in the oat drink. Given that oat drink processing (e.g., enzymatic treatment, molecular weight reduction) can substantially affect β-glucan viscosity and biological activity, the estimated intake may not reflect biologically effective β-glucan [40].

Participants were predominantly from high socioeconomic backgrounds, which may limit the applicability of this study to broader populations. Additionally, relying on two 24-HR may not accurately reflect habitual intake or capture subtle dietary shifts. Despite these limitations, the study provides valuable preliminary data and highlights the need for larger, longer-term trials with more rigorous dietary assessment and mechanistic endpoints. Further research should also explore the environmental impact of switching from cow’s milk to oat drink in the diet.

## Conclusion

Daily consumption of 600 mL of oat drink over four weeks resulted in a modest but statistically significant reduction in total cholesterol and LDL-C compared with cow’s milk in healthy young women. No significant differences were observed for HDL-C, triglycerides, or body weight. The total cholesterol and LDL-C reduction likely reflects a combination of β-glucan intake and improved dietary fat quality, rather than changes in energy intake or body weight. While these findings are promising and the intervention was feasible with high compliance, the findings should be interpreted as exploratory, and further research is warranted to confirm efficacy and inform dietary recommendations.

## Supplementary Information

Supplementary Material 1.

Supplementary Material 2.

## Data Availability

De-identified data can be shared upon request to the first author.
