# Supplementary material for: Cow’s milk compared to oat drink and its implications for lipid profile– a pilot randomized controlled trial
Source: Nutr J. 2026 Mar 18;25:54. doi: 10.1186/s12937-026-01314-w (PMC13112611; doi:10.1186/s12937-026-01314-w)
Supplement: Supplementary file 1 — Supplementary Material 1. [file 12937_2026_1314_MOESM1_ESM.docx]

**Supplementary Figure 1.** Consolidated Standards of Reporting Trials (CONSORT )2010 Flow Diagram illustrating the flow of the participant through the pilot RCT. A total of 77 women were assessed for eligibility. Of these, 44 were excluded: 2 did not meet the inclusion criteria, 21 were not reached after initial contact, 9 declined due to inconvenience, 4 declined due to study burden, and 8 did not provide a reason. The remaining 33 participants signed the consent form, were enrolled in the trial and completed the first study visit. They were randomized into either the intervention arm (n=17), or the control arm (n=16). One participant in the control arm did not complete the trial and was excluded from final analyses.
