# Supplementary material for: Cow’s milk compared to oat drink and its implications for lipid profile– a pilot randomized controlled trial
Source: Nutr J. 2026 Mar 18;25:54. doi: 10.1186/s12937-026-01314-w (PMC13112611; doi:10.1186/s12937-026-01314-w)
Supplement: Supplementary file 2 — Supplementary Material 2. [file 12937_2026_1314_MOESM2_ESM.docx]

Supplementary table 1: TSH, fT4, fT3, Tg and TPOAb at baseline and end of study in the participants in the milk-study (total participants n=32)

|  | **Baseline** |  | **End-study** |  | **Outcome differences p** | |
| --- | --- | --- | --- | --- | --- | --- |
|  | **median (p25-p75)** |  | **median (p25-p75)** |  | **p-value^a^**  within groups, (baseline to end of study) | **p-value^b^** between groups |
| **TSH (mIU/L)** |  |  |  |  |  |  |
| All participants (n=32) | 1.3 (1.0-1.8) |  | 1.4 (1.2-2.0) |  | 0.027 |  |
| Oat drink (n=17) | 1.4 (0.9-2.0) |  | 1.3 (1.2-1.8) |  | 0.475 | 0.140 |
| Cow’s milk (n=15) | 1.3 (1.2-1.8) |  | 1.6 (1.1-2.1) |  | 0.026 |  |
| **fT4 (pmol/L)** |  |  |  |  |  |  |
| All participants (n=32) | 15.8 (14.3-16.7) |  | 14.8 (14.0-16.5) |  | 0.110 |  |
| Oat drink (n=17) | 16.0 (14.7-16.7) |  | 15.1 (14.3-16.7) |  | 0.477 | 0.584 |
| Cow’s milk (n=15) | 14.7 (14.1-16.8) |  | 14.6 (13.6-15.8) |  | 0.158 |  |
| **fT3 (pmol/L)** |  |  |  |  |  |  |
| All participants (n=32) | 4.7 (4.0-5.1) |  | 4.6 (4.2-5.0) |  | 0.428 |  |
| Oat drink (n=17) | 4.7 (4.0-5.1) |  | 4.7 (4.0-5.0) |  | 0.442 | 0.705 |
| Cow’s milk (n=15) | 4.7 (4.1-4.9) |  | 4.5 (4.3-5.4) |  | 0.649 |  |
| **Tg (µg/L)** |  |  |  |  |  |  |
| All participants (n=32) | 13.9 (9.1-20.6) |  | 11.9 (8.5-21.5) |  | 0.688 |  |
| Oat drink (n=17) | 9.5 (7.1-14.3) |  | 9.3 (7.6-12.4) |  | 0.244 | 0.121 |
| Cow’s milk (n=15) | 18.5 (13.9-24.0) |  | 20.0 (11.6-25.0) |  | 0.490 |  |
| **TPOAb (kIU/L)** |  |  |  |  |  |  |
| All participants (n=32) | 10.0 (9.5-13.0) |  | 11.5 (9.9-14.0) |  | 0.368 |  |
| Oat drink (n=17) | 10.0 (9.1-13) |  | 11.0 (9.6-13.0) |  | 1.000 | 0.190 |
| Cow`s milk (n=15) | 10.0 (10-14) |  | 12.0 (11.0-14.0) |  | 0.304 |  |

| **Supplementary table 2.** The difference in urinary iodine concentration (UIC), urinary iodine excretion (UIE), estimated daily iodine intake from 24-hour dietary recall and estimated daily iodine intake derived from UIE, from baseline to end of study and between the two groups, oat drink and cow’s milk, in women in the milk study (n=32) | | | | | | | | | | | | | |
| --- | --- | --- | --- | --- | --- | --- | --- | --- | --- | --- | --- | --- | --- |
|  |  | **Baseline** | | |  | **End-study** | | |  | **Outcome difference** | | |  |
|  |  | **Median** | **(p25-p75)** | **min, max** |  | **Median** | **(p25-p75)** | **min, max** |  | **p-value^b^**  baseline to end of study | **p-value^c^**  between groups |  |  |
| **UIC (µg/L)ª** |  |  |  |  |  |  |  |  |  |  |  |  |  |
| All participants (n=32) | | 51 | (42-62) | 20, 120 |  | 66 | (61-79) | 41, 710 |  | <0.001 |  |  |  |
| Oat drink (n=17) | | 49 | (35-76) | 29, 120 |  | 66 | (62-80) | 41, 100 |  | 0.008 | 0.485 |  |  |
| Cow’s milk (n=15) | | 54 | (49-66) | 20, 84 |  | 70 | (55-77) | 47, 710 |  | 0.002 |  |  |  |
| **UIE (µg/24h)** |  |  |  |  |  |  |  |  |  |  |  |  |  |
| All participants (n=32)^d^ | | 128 | (104-157) | 46, 426 |  | 162 | (133-196) | 64, 611 |  | <0.001 |  |  |  |
| Oat drink (n=17) | | 123 | (97-143) | 43, 426 |  | 147 | (133-179) | 64, 299 |  | 0.076 | 0.721 |  |  |
| Cow’s milk (n=15)^d^ | | 137 | (112-174) | 70, 195 |  | 180 | (136-207) | 64, 611 |  | 0.003 |  |  |  |
| **Estimated intake from UIE (µg/24h)** | |  |  |  |  |  |  |  |  |  |  |  |  |
| All participants (n=32)^d^ |  | 143 | (115-174) | 51, 473 |  | 180 | (148-218) | 71, 678 |  | <0.001 |  |  |  |
| Oat drink (n=17) |  | 136 | (108-156) | 51, 473 |  | 163 | (148-198) | 71, 332 |  | 0.076 | 0.204 |  |  |
| Cow’s milk (n=15)^d^ |  | 152 | (124-193) | 77, 217 |  | 200 | (152-230) | 71, 678 |  | 0.003 |  |  |  |
| **Estimated intake from 24h dietary recall (µg/d)** | |  |  |  |  |  |  |  |  |  |  |  |  |
| All participants (n=32) | | 126 | (94-165) | 27, 281 |  | 175 | (135-262) | 76, 840 |  | <0.001 |  |  |  |
| Oat drink (n=17) |  | 128 | (103-189) | 68, 252 |  | 169 | (131-249) | 109, 407 |  | 0.008 | 0.079 |  |  |
| Cow’s milk (n=15) |  | 126 | (60-152) | 27, 281 |  | 199 | (144-284) | 76, 870 |  | 0.002 |  |  |  |
| ªUIC (µg/L) was analysed from 24-h urine iodine excretion sample (UIE)  ^b^Assessed by Wilcoxon signed ranks test.  ^c^Assessed by Mann-Whitney U test.  ^d^At end of study, n=31 for all participants and n=14 for the cow’s milk group due to missing 24h urine excretion volume data. A two-sided p-value of <0.05 was considered statistically significant.  UIC, urinary iodine concentration; UIE, urinary iodine excretion; n, number of participants; r. | | | | | | | | | | | | | |
|  |  |  |  |  |  |  |  |  |  |  |  |  |  |
|  |  |  |  |  |  |  |  |  |  |  |  |  |  |
|  |  |  |  |  |  |  |  |  |  |  |  |  |  |
